# Effectiveness of Remote Intensive Counseling Versus Outpatient Counseling in Substance Use Disorders: A Retrospective Cohort Study

**DOI:** 10.7759/cureus.24167

**Published:** 2022-04-15

**Authors:** Armand Ntchana, Ricky Daley

**Affiliations:** 1 Family Medicine, Oceania University of Medicine, McAllen, USA; 2 Urology, United States Navy, Annapolis, USA

**Keywords:** tele health, substance use, relapse, prevention, effectiveness

## Abstract

Background

Substance use disorders are a serious and persistent U.S. public health problem. Although a number of therapy modalities exist, few studies assessed the comparative effectiveness of specific therapies. This study empirically evaluated whether remote intensive counseling (RIC) is more effective than outpatient therapy (OT) in relapse prevention over the period of nine months in patients aged 18-45 years with a history of substance use.

Methods

The current study utilized a retrospective correlational cross-sectional cohort quantitative research design with multiple between-group comparisons and fixed effects. The sample (n=296) included adults of both sexes, of diverse racial/ethnic backgrounds, and of socioeconomic status (SES) between 18-45 years of age who had been using an illicit addictive substance(s) for at least six months prior and had never participated in any treatment program previously. Individuals with alcohol and/or nicotine co-dependence were excluded.

Result and conclusion

Remote intensive counseling (RIC) is more effective for patients aged 18-45 years with a history of substance use than outpatient therapy (OT). RIC works better for single or never married females younger than 30-year-old with higher education. The use of RIC for other age and racial/ethnic groups should be guided by whether patients belong to a younger age cohort and/or a specific race/ethnicity.

## Introduction

Background

Various substance use disorders and especially recently rampant prescription opioid misuse, abuse, and addiction epidemic have reached enormous proportions and become serious and persistent U.S. public health problems. Nonmedical use of prescription drugs affects >2.1M people annually [1-4]. Death reports vary by source but are nonetheless shocking - according to the President’s Commission on Combating Drug Addiction and the Opioid Crisis, occasional drug use (ODU) is responsible for 143 deaths a day, while opioid-related deaths resulting from misuse and overdose now exceed the murder rate by the gun [5].

Over 30K/year die from opioids, while fentanyl is responsible for nearly 10K deaths a year [6]. Channels of illicit distribution are rapidly multiplying and the customer base is growing fast [7]. Overall, as a class of drugs, opioids are responsible for 34K/year overdose deaths in the United States [8]. The economic costs are equally high. The National Drug Intelligence Center estimated that costs of illicit substance abuse are over $193 billion annually in costs related to crime, lost work productivity, and healthcare [9]. However, some recent estimates of the combined economic burden of both illicit and prescription opioids suggest that the real cost of the U.S. drug epidemic may in reality approach $1 trillion, especially when the loss of productivity and indirect social costs are fully considered [10].

Society has tried to combat this horrific epidemic with an array of institutional interventions intended to stop, prevent, and mitigate its consequences: (a) prescription drug monitoring programs, (b) state laws regulating sales or use of prescription drugs [11], (c) stringent insurance requirements and screening tests, (d) school and community-based programs [12], (e) patient education efforts [13], and (f) increased law enforcement (e.g., drug courts with mandatory treatment [14,15].

However, interventionists confront multiple causative factors such as inappropriate prescribing and monitoring, misuse and self-medication, pill sharing, “doctor shopping” to obtain redundant prescriptions, illegal sales, and other ecological factors [16,17]. These factors extend the risks already known to predispose individuals to abuse and addiction - prior use [18], incarceration [19], and concomitant alcoholism [20-22].

Although many risk factors are not fully avoidable, success in treatment and recovery may depend on a better understanding of populations at high risk of drug addiction before they become victims, especially those with prescription opioid misuse, abuse, and addiction. It is also critical to get a better understanding of what programs and clinical interventions may work best for specific affected populations.

Currently, a number of treatment modalities can be used for various substance use disorders. They include individual and group counseling, inpatient and residential treatment, outpatient therapy, partial hospital programs, case and care management, medication, recovery support services (including community-based programs), 12-step fellowship, and peer support. As mobile communication and IT (information technology) have evolved in recent years, remote intensive counseling has also been utilized as a treatment modality [23-25].

However, even more, important in this challenging context is to have the most current and objective data on the comparative effectiveness of various treatments and interventions. Thus, the purpose of the current study was to evaluate empirically whether remote intensive counseling (RIC) was more effective than outpatient therapy (OT) in relapse prevention over the period of nine months in patients aged 18-45 years with a history of opioid substance use.

Review of the literature

Substance use and illegal prescription drug use affect many communities across the United States. Individuals aged 18 to 45 years are most adversely affected by this crisis [26]. While alcohol and tobacco are the two substances used most frequently by this population group, they are directly followed by marijuana, and other illicit drugs [27]. However, in recent years, prescription opioid misuse, abuse, and addiction have become more prevalent [11,15].

While the incidence of substance use among Americans 18 years and older has been steadily decreasing over the last 15 years, nevertheless, an estimated 24.6M or 9.5% of Americans in this age group had used an illicit drug in the past month, and this number is up from 8.4% in 2003, which is a reflection of a recent rise in use of marijuana, the most commonly used illicit drug [4]. After alcohol, cannabinoids are associated with the highest rate of dependence or abuse - 4.3M users, which is five times the number of cocaine dependents [4].

What is even more alarming is that an estimated 22.9M Americans or roughly 8.7% needed treatment for a problem directly related to substance abuse but only about 2.6M of people or less than 1.1% affected received professional treatment or therapy. The statistics for the age group 18-45 years are equally bad [4]. The existence of such a large treatment gap and the disturbing substance abuse statistics among this population cohort indicate the urgent need for therapies that can be both effective and cost-efficient.

In this context, the research problem of this study was two-dimensional. On the one hand, people struggling with substance misuse, abuse, and addiction do not have proper access to the care they need to prevent a relapse (i.e., treatment gap). On the other hand, treatment options, which are currently available to patients, may not meet all requirements of the complex, dynamic and evolving demographics most affected by this problem.

With recent rapid advances in IT available for clinical use [28,29], and especially for the purposes of substance abuse treatment [30,31], new approaches have emerged including RIC [26]. It remains unclear whether RIC is as effective as OT in relapse prevention in patients 18 years and older suffering from opioid misuse, abuse, and addiction. We conducted a selective review of the literature on the topic of RIC effectiveness and the findings are the following.

First, while substance misuse, abuse and addiction, and effective therapies are an active research stream, studies that investigated RIC remain scarce. Such paucity may be explained by the fact that RIC is a relatively novel approach, which may not be practiced by many providers [32]. Alternatively, it may be that the very IT tools necessary for its successful implementation by clinical practitioners may have not penetrated the counseling practice to achieve sufficient levels of technological saturation [33].

Second, the evidence presented is predominantly patient-oriented rather than disease-oriented [34]. Most studies focus on relevant clinical outcomes of either general or specific importance to substance abuse patients, such as changes in morbidity and comorbidity, effects of specific treatments on quality of life, the efficacy of relapse prevention, comparative effectiveness of therapies, etc. Only in some studies, disease-oriented evidence was reported [35,36].

Third, in terms of topics, past research focuses on areas of comparative effectiveness of various therapies [37], optimality of specific interventions [38], mediating variables [25,36], clinical approaches to relapse prevention in substance abuse patients [39,40], the efficacy of specific therapies particular populations [41-44]. Only a few studies have evaluated the effectiveness of RIC per se [24,32,45-47].

Fourth, substantively extant clinical research suggests that in terms of comparative effectiveness, family therapy, mixed group therapy, and cognitive behavioral therapy are generally more effective in reducing substance abuse dependence in this population group [48,49]. In contrast, cognitive behavioral therapy is effective as monotherapy and as a part of combination therapy in this clinical demographic [50].

Fifth, the effectiveness of any form of substance abuse therapy in patients 18-45 years can be enhanced by community-based continuing care that is equally effective for outpatient and remote modalities [35,44] but moderated by patients’ socioeconomic status [38], access to services [32,51], and duration of the therapy [37]. Studies of RIC also found that it can be as effective as face-to-face approaches [32,40,45,47], but for optimal outcomes, it should be closely integrated with support services [39,45].

Overall, this evidence suggests that the effectiveness of RIC vs. OT in relapse prevention in patients 18 years and older is largely unaddressed by extant research and merits further investigation. The current study investigated this topic and its findings contribute to the growing body of knowledge on whether RIC is clinically effective and cost-efficient.

## Materials and methods

Design

The study utilized a retrospective correlational cross-sectional cohort quantitative research design with multiple between-group comparisons and fixed effects. No variables in the study were manipulated. Remote intensive counseling (RIC) is defined as a Telemental Health (TMH) modality in which a trained psychotherapist meets with a client via telephone, mobile phone, or the internet in place of or in addition to conventional psychotherapy [33]. Outpatient therapy (OT) is defined as a treatment modality structured so that patients have freedom of movement to maintain a regular commitment to family, work, and education [52]. Relapse is defined as a single incidence of compulsive return to substance use [53,54].

Data

The study used depersonalized individual-level data collected in 2016-2017 for an unimplemented public health study in Connecticut that was intended to explore specific factors associated with substance use among participants aged 18-65 years. The authors of the dataset have provided their permission to use the data. Data were collected using reports from participating therapists. The final analytical dataset was derived by excluding all research participants beyond 18-45 years of age. Such cut-off criterion is based on two considerations: (a) the majority of drug addiction therapies are focused on adults [41,55], and (b) because one of the therapies implies extensive reliance on remote IT, adults older than 45 years of age have lower IT proficiency, and may involuntarily introduce a certain degree of response bias that may contaminate the results [56]. The dataset contains multiple observations of some sociodemographic measures and on the incidence of relapses during nine months of the study observed at three points of observation (respectively at three, six, and nine months).

Population and sample

The population included adults of both sexes, of diverse racial and ethnic backgrounds between 18 and 45 years of age, and of various socioeconomic statuses. The inclusion criteria were: (a) participant had been using an illicit addictive substance(s) (e.g., opioids, cannabinoids, cocaine, methamphetamines, etc.) for at least six months prior to therapy, and (b) participant had never participated in any treatment program previously. Individuals who were also codependent on alcohol and/or nicotine were excluded from the sample.

The participants had taken either remote intensive counseling (RIC) or outpatient therapy (OT). The treatments were not standardized and were delivered by a licensed addiction counsel at least twice weekly for the period of nine months. This measure allowed to achieve efficiency in the size of the patient sample needed to compare the effectiveness of both interventions in regard to their effects on relapse prevention within the nine months of the study. The participants remained in the cohort after any single incidence of relapse.

Analyses

The study was designed with a reasonable expectation that a certain measurable probability does exist that would provide a definitive answer to the question of whether remote intensive counseling (RIC) is more effective in relapse prevention than outpatient therapy (OT) in patients with substance misuse, abuse, and addiction in nine months. Statistically, the study compared the effectiveness of two treatments (OT vs. RIC) with respect to the incidence of failures (i.e., relapses) observed in each group of participants. The repeated measures ANCOVA was used to assess the differences between groups on a single dependent variable (relapses) after controlling for the effects of covariates (education, income, age, race, etc.). The F-test of significance was used to assess for differences.

Due to the expected outcomes of the analysis of covariance (ANCOVA), two additional statistical procedures were performed. Principal component analysis (PCA) was utilized to determine the factors contributing the most to relapses in specific groups. Time series (TS) were utilized to determine the effects of the two approaches over time (t = nine months) and to identify any moderating and mediating effects of covariates. The PCA and TS were needed because this study aimed to detect a rather weak signal (difference in the effects of treatment) in a very noisy environment (inevitable patient variability caused by prognostic factors, adherence to therapies, etc.). The study was designed to exclude any typical confounders and biases.

## Results

The response rate was high among 413 participants who completed the original survey. The final analytical sample included 296 participants (Table 1).

**Table 1:** Demographics of participants by groups. RIC: remote intensive counseling; OT: outpatient therapy; GED: general educational development

Characteristics	OT (n = 153) (%)	RIC (n = 143) (%)
Gender		
Female	57 (37.2)	72 (50.3)
Male	96 (62.8)	71 (49.7)
Age (years), mean (SD)	29 (9.1)	31 (9.2)
Age group (years)		
18-30	93 (60.7)	69 (48.3)
31-35	9 (5.8)	19 (13.3)
36-45	51 (33.5)	55 (38.4)
Education		
< High school	22 (14.4)	15 (10.5)
High school/GED	60 (39.2)	55 (38.5)
Associate degree	32 (20.9)	28 (19.6)
≤ Bachelor's degree	39 (25.5)	45 (31.4)
Income, $1000		
< $25	44 (28.7)	31 (21.7)
$25-$50	59 (38.6)	58 (40.6)
$50-$75	38 (24.8)	39 (27.2)
$75+	12 (7.9)	15 (10.5)
Race/ethnicity		
Native American	2 (1.3)	2 (1.4)
Asian American	9 (5.9)	9 (6.3)
African American	60 (39.2)	56 (39.2)
Hispanic/Latino	31 (20.3)	26 (18.2)
White	51 (33.3)	50 (34.9)
Marital status		
Single/never married	72 (47.1)	60 (41.9)
Married/partnered	69 (45.1)	68 (47.6)
Separated/divorced	12 (7.8)	15 (10.5)
Incidence of relapses		
t = 3 months	106 (69.3)	94 (65.7)
t = 6 months	49 (32.1)	36 (25.2)
t = 9 months	17 (11.1)	8 (5.6)

Analysis of covariance

ANCOVA tests determined whether there were statistically significant differences in relapses between the two groups (RIC vs. OT) after controlling for sex, age, income, race/ethnicity, and marital status (Table 2). In all analyses, both OT and RIC had significant effects on the incidence of relapses among the participants after controlling for covariates. In three analyses, the effects of RIC were stronger than the effects of OT, especially after controlling for sex F-test for OT (FOT) (1, 148) = 17.33 < F-test for RIC (FRIC) (1, 138) = 19.89, after controlling for age FOT (1, 148) = 13.19 < FRIC (1, 138) = 14.01, and after controlling for marital status FOT (1, 148) = 4.56 < FRIC (1, 138) = 5.04. In two analyses, after controlling for education (FOT {1, 148} = 4.61 ≈ FRIC {1, 138} = 4.56) and after controlling for income (FOT {1, 148} ≈ 21.17 < FRIC {1, 138} = 21.2), OT and RIC exerted approximately similar effects. Finally in one analysis, after controlling for race/ethnicity, OT had comparatively stronger effects than RIC (FOT {1, 148} = 7.68 > FRIC {1, 138} = 6.13).

**Table 2:** ANCOVA between groups effects. ^a^All variables are ln transformed; α = 0.05. ^b^df = 1. RIC: remote intensive counseling; OT: outpatient therapy

ANCOVA between groups effects^a^			
Group	Factors^b^	F	p > F
OT (n = 153)	Sex	17.33	< 0.0001
	Age	13.19	< 0.0001
	Education	4.61	< 0.0001
	Race/ethnicity	7.68	< 0.0001
	Income	21.17	< 0.0001
	Marital status	4.56	< 0.0001
RIC (n = 143)	Sex	19.89	< 0.0001
	Age	14.01	< 0.0001
	Education	4.56	< 0.0001
	Race/ethnicity	6.13	< 0.0001
	Income	21.2	< 0.0001
	Marital status	5.04	< 0.0001

Principal component analysis

Because of mixed results of the ANCOVA, principal component analysis (PCA) was conducted on each group (Table 3). The results suggest that different factors contribute to the maximal amount of explained variance in the likelihood of relapse in each group. While sex, age, race/ethnicity, and education were cumulatively responsible for 62% of explained variation in the incidence of relapses in the OT group, income, and marital status were cumulatively responsible for only 15% of explained variation. The picture is different for the group that received RIC intervention - factors of age, education, and income accounted for almost 50% of explained variation. Marital status, sex, and race/ethnicity contributed an approximately equal amount of explained variation in the incidence of relapses in this group. Overall, these differences suggest that the same sociodemographic characteristics may act as either mediators or moderators for RIC and OT.

**Table 3:** Principal component analysis. RIC: remote intensive counseling; OT: outpatient therapy

Group		Eigenvalue	Δ	Proportion	Cumulative
OT (n = 153)	Sex	5.34	2.97	0.31	0.31
	Age	2.37	0.59	0.14	0.45
	Race/ethnicity	1.78	0.30	0.10	0.56
	Education	1.48	0.29	0.09	0.62
	Income	1.19	0.19	0.07	0.72
	Marital status	1.00	0.06	0.06	0.77
RIC (n = 143)	Age	6.11	3.18	0.37	0.37
	Education	3.28	1.01	0.13	0.40
	Income	1.64	0.27	0.09	0.49
	Marital status	1.13	0.17	0.06	0.55
	Sex	1.07	0.10	0.05	0.60
	Race/ethnicity	1.02	0.08	0.04	0.64

Time series

Time series analysis was conducted to determine the effects of the two therapies over time (t = nine months) and to examine the effects of covariates. The results are shown in Table 4. While both RIC and OT contribute to reduction in the incidence of relapses over time (t = nine months), when assessed comparatively RIC is a more effective intervention (2.7% vs. 1.1% reduction, respectively). The covariates exert differential effects on the treatment outcomes in the two groups, sex appears to have a mediating effect on the outcome of both therapies.

**Table 4:** Time series analysis. ^a^α = 0.05. RIC: remote intensive counseling; OT: outpatient therapy

Time series analysis^a^		
Variables	OT (n =153)	RIC (n = 143)
Relapses	-0.011	-0.027
Female	-0.012	-0.026
Male	-0.01	-0.014
Age group (years)		
18-30	-0.141	-0.183
31-35	-0.101	-0.147
36-45	0.036	0.031
Education	-0.014	-0.057
Income	0.01	0.01
Race/ethnicity		
Native American	n/a	n/a
Asian American	n/a	n/a
African American	0.016	0.008
Hispanic/Latino	0.012	0.012
White	0.113	0.111
Marital status		
Single/never married	-0.011	-0.041
Married/partnered	0.007	0.003
Separated/divorced	0.009	0.011
$\bar{R}^{2}$	0.83	0.85

Females respond better to either RIC (2.6% vs.1.4%) or OT (1.2% vs.1.0%), and in terms of comparative effectiveness, with RIC females demonstrate better outcomes by ≈1.2% but only marginally better outcomes by 0.2% with OT. Belonging to a specific age group has differential effects on the effectiveness of the two therapies. While the effectiveness decreases with age group with both RIC and OT, younger clients respond better to both interventions but especially to RIC (18.3% vs. 14.1% in < 30 years old and 14.7% vs. 10.1% in < 35 years old).

Education is a moderating factor but it plays a stronger role in the effectiveness of the RIC (5.7% vs. 1.4%). Income appears to have a general moderating effect on the effectiveness of both interventions but its effects were largely marginal in both cases (≈1%). A more nuanced picture is observed with race. For all three race groups in the study, race had a moderating effect on the effectiveness of both RIC and OT. However, while overall African Americans and White responded better to RIC than OT, for Hispanics no difference in the effects was observed.

The effects of marital status on the outcomes of both treatments were mixed. Single or never married participants responded better than the participants with another marital status to both interventions, but RIC appears to work better for them (4.1% vs. 1.1%) than OT. In contrast, being married/partnered or separated/divorced/widowed acted as a moderator for both therapies with differential effects in each case. This is surprising because the literature suggests that being single typically has adverse effects on the outcomes of substance use intervention [1,11].

## Discussion

In this study, the effectiveness of remote intensive counseling (RIC) was compared to outpatient therapy (OT) in relapse prevention over the period of nine months in patients aged 18-45 years with a history of substance misuse, abuse, and addiction. Overall, the results of statistical analyses provide credible empirical evidence that remote intensive counseling (RIC) is a more effective therapeutic intervention for patients with a history of substance use than outpatient therapy (OT), at least in the cohort of patients aged 18-45 years.

Extant literature that explored the behaviors and coping mechanisms that are most commonly associated with the risk of addiction concluded that not only individual genetic predisposition and personality traits (e.g., impulsive and compulsive behaviors, nonconformity, low self-esteem, lack of patience, denial, antisocial behavior, suggestibility, etc.) may lead to the development of addiction, especially drug addiction [35,57-59] but also general socioeconomic characteristics of susceptible individuals may act as either mediators or moderators of addiction [1,12,16]. Therefore those should be factored into the design and evaluation of effective and cost-efficient therapeutic interventions [14,15,19].

In this context, the results of the current study confirm the latter claim. They also underscore the need to reconceptualize the addiction development process and properly account for all contributing factors that may play a significant role in it. Furthermore, because the current study empirically demonstrated that different sexes, racial, and age groups respond differentially to the same and also to different interventions in terms of their therapeutic outcomes, it is critical to address such differential effects from the practical viewpoint, i.e., adapt existing and design new therapeutic approaches so that moderating effects of socioeconomic characteristics are accurately mitigated, while mediating effects are adequately amplified.

Remote intensive counseling (RIC) remains a relatively novel therapeutic approach, especially in the form that relies on mobile applications and wearable sensors. The results of the current study suggest that RIC generally works best for single/never married females < 30 years old with higher levels of educational attainment. This specific group of patients is more likely to benefit from the RIC. The use of the RIC for other age and racial groups should be guided by whether they belong to a younger age group and/or a specific racial/ethnic group. While the results of this study may not be fully generalizable, they still provide a solid rationale for decision-making regarding the use of specific therapeutic interventions.

The current study has several limitations. In the original dataset, the treatments were not standardized and the selected approach to experimental mortality (i.e., participants remained in the cohort even after an incidence of relapse, some had multiple relapses) to some degree weaken the internal validity of the current study. Also, extant studies suggest that substance use history is one of the strongest predictors of treatment outcomes [6,11, 12,19,20]. Regrettably, this information was not available. However, clinical studies suggest that different addictive substances have dissimilar reactive effects in terms of the severity, duration, and outcomes of addiction [54-58,59] as well as on the probabilities of recovery [41]. Because these factors are important but not accounted for in this study, they adversely affect its external validity. Future studies on this topic must include data on participants’ substance use histories and the specific types of substances used. The scope and scale of future investigations of the comparative effectiveness of OT and RIC and other addiction treatment modalities (eye movement desensitization and reprocessing {EMDR}, cognitive behavioral therapy {CBT}, dialectical behavior therapy {DBT}, etc.) should also be increased.

The results have several implications for practice, education, and policy. The results can be used to increase the efficacy of substance use counseling practice. In many cases, substance use counselors do not have sufficient guidance to make an informed evidence-based decision regarding the choice of a particular therapeutic intervention for a client [20,23]. The findings of this study allow substance use counselors not only to decide on the appropriateness of RIC or OT but tailor them based on the client’s individual background, history of substance use, and recovery needs. Additionally, our results may be used to inform the educational content of substance use counseling programs. Because our results demonstrate that different socioeconomic characteristics of patients have differential effects on the intervention outcomes, this should contribute to a meaningful review of the current programs, adjustment of the recommended clinical pathways, and alteration of available approaches to addiction case management.

## Conclusions

The results overall support the claim that remote intensive counseling is a comparatively better choice of therapeutic intervention in patients aged 18-45 years. The utilization of RIC as a new treatment modality should be expanded. RIC should become an intervention of choice for the populations that benefit the most from it. Such expansion must be reflected in the federal and state policies that permit the use of such treatment modalities. This should also lead to a substantive revision of reimbursement policies and a change in the level of reimbursements.
